# Cardiovascular disease events and its predictors in women: Isfahan Cohort Study (ICS)

**DOI:** 10.15171/jcvtr.2017.27

**Published:** 2017-08-21

**Authors:** Masoumeh Sadeghi, Azam Soleimani, Hamidreza Roohafza, Safoura Yazdekhasti, Shahram Oveisgharan, Mohammad Talaei, Nizal Sarrafzadegan

**Affiliations:** ^1^Cardiac Rehabilitation Research Center, Cardiovascular Research Institute, Isfahan University of Medical Sciences, Isfahan, Iran; ^2^Heart Failure Research Center, Cardiovascular Research Institute, Isfahan University of Medical Sciences, Isfahan, Iran; ^3^Isfahan Cardiovascular Research Center, Cardiovascular Research Institute, Isfahan University of Medical Sciences, Isfahan, Iran; ^4^Shariati Hospital, Tehran University of Medical Sciences, Tehran, Iran; ^5^Rush Alzheimer’s Disease Center, Rush University Medical Center, Chicago, IL, USA; ^6^Saw Swee Hock School of Public Health, National University of Singapore, Singapore

**Keywords:** Women, Ischemic Heart Disease, Stroke, Risk Factor

## Abstract

***Introduction:*** As a lack of validated data about cardiovascular (CV) events and its risk factors (RFs) in women of Eastern Mediterranean region, we aimed to evaluate common predictors of CV events among Iranian women.

***Methods:*** Isfahan cohort study (ICS) is a prospective cohort that followed 6323 residents (51.3% women, aged 35-75 years) from three counties and their rural districts in central Iran. Common cardiovascular disease (CVD) RFs namely hypertension (HTN), diabetes mellitus, dyslipidemia, abdominal obesity, smoking, low apolipoproteins A ( apo-A) and high apolipoprotein B (apo-B) were evaluated. End points (CV events) were defined as fatal and nonfatal myocardial infarction, sudden cardiac death (SCD), unstable angina and stroke.

***Results:*** After 9 years of follow-up, 265 CV events were detected. The mean age of women with CV event was 57.6±10.9; about 8 years older than those without event. All CV RFs were significantly more prevalent in women with CV event except for low HDL cholesterol, overweight and low apo-A. HTN, diabetes, high triglyceride (TG), high LDL-C and obesity were significantly associated with CV events after adjustment for age, smoking and menopausal status (hazard ratios [95% CI]: 2.56 [1.93, 3.95], 2.43 [1.76, 3.35], 2.02 [1.49, 2.74], 1.59 [1.20, 2.11] and 1.49 [1.16-1.92], respectively), while low HDL cholesterol and abdominal obesity were not predictors for CV events (hazard ratios [95% CI]: 1.26 [0.96, 1.65], 1.71 [0.99, 2.96], respectively).

***Conclusion:*** In ICS, HTN, diabetes mellitus and high triglyceride are strong predictors for CV events in Iranian women. As almost all strong risk markers of CVD events are preventable, health policy makers have to give urgent consideration to make preventive public health strategies.

## Introduction

Cardiovascular disease (CVD) is the leading cause of death in both men and women^1^ and it is estimated that with elimination of all forms of major CVD, life expectancy would increase by almost seven years.^2^ In Middle Eastern countries, CVD is the main cause of mortality and is responsible for 42% of all deaths, that 47% of them were coronary heart disease, in 2010.^2-4^ Middle East is currently in the third to fourth phase- the phase of degenerative and man-made disease and delayed degenerative disease- of epidemic transition.^2^ It is estimated that by the year 2025, 80%-90% of all CVD events will occur in low and middle-income countries^3^ which indicate the necessity of strategies for identification and control of more power full cardiovascular risk factors (RFs) in each country.

Although CVD is the cause of mortality in one third of women and when occurs, it is more fatal than men, women are sub group that are underrepresented in most clinical studies.^4^ Moreover, RFs and clinical manifestations of CVD may be different from those observed in men.^5^ Several studies evaluated common cardiovascular RFs in developed countries including known modifiable traditional RFs such as diabetes mellitus, hypertension (HTN), smoking, obesity, dyslipidemia and some novel RFs such as hsCRP, inflammatory markers (interleukins), lipoprotein a, apo A, apo B and their ratios^6,7^ but still there is a lack of sufficient data about effect of common RFs on cardiovascular events in Middle East women.

To address these issues, as a part of Isfahan Cohort Study (ICS) we evaluated the prevalence of common cardiovascular RFs and their effects on CV events in women of urban and rural areas of three central counties in Iran.

## Materials and Methods

### Subjects

The ICS^8^ is an ongoing longitudinal community-based study of 6323 adults aged 35 years and older at baseline, living in both urban and rural areas of three counties in the central Iran (Isfahan, Arak and Najafabad). Cohort study population had participated in IHHP (Isfahan Healthy Heart Program) baseline survey^9^ which is a community trial for CVD prevention and control. Details of ICS methodology have been previously reported.^8^ Briefly, the participants were recruited from January 2 through September 28, 2001 based on multistage random sampling. Our exclusion criterion at baseline was the history of CVD and the remaining subjects have been followed since then.

### Data collection

Data collection including medical interview, physical examination and laboratory assays was conducted by trained health personnel by using a validated questionnaires, calibrated instruments and standard protocol as has described previously.^8^ Dyslipidemia was defined as if low density lipoprotein-cholesterol (LDL-C) ≥130 mg/dL, total cholesterol (TC) ≥200 mg/dL, triglycerides (TG) ≥150 mg/dL or high density lipoprotein-cholesterol (HDL-C) <40 mg/dL in men or <50 mg/dL in women. Diabetes mellitus was defined as if fasting blood glucose ≥126 mg/dL or the patient was receiving anti-diabetic agents; impaired glucose tolerance was identified if the 2-hour postprandial blood glucose ≥140 but less than 200 mg/dL. The body mass index (BMI) value of 25-29.9 kg/m^2^ was classified as overweight and those who had BMI ≥30 kg/m^2^ were classified as obese. According to International Diabetes Federation cut points, waist circumference (WC) ≥94 cm in men or ≥80 cm in women were defined as abdominal obesity. Waist-to-hip ratio ≥0.95 in men and ≥0.8 in women was considered as a high waist-to-hip ratio. Patients with two readings of blood pressure ≥140/90 mm Hg or anti-hypertensive drug consumers were classified as HTN. Participants who used at least one cigarette per day were considered as current smokers.

### Follow up

All subjects were followed biannually for occurrence of cardiovascular events (CV events), including ischemic heart disease (fatal and nonfatal myocardial infarction [MI], sudden cardiac death [SCD], and unstable angina [UA]) or stroke. This paper is based on the ninth years of follow up in 2011.

To confirm events two separate panel of four cardiologists and neurologist who were blinded to the subjects’ RF profiles reviewed all relevant documents including death certificates and verbal autopsy to make a final decision. The occurrence of myocardial infarction was confirmed if symptoms met the criteria of the World Health Organization.^10^ Acute MI was diagnosed if two of three following criteria were observed: (1) typical chest pain lasting more than 30 minutes, (2) ST elevation ≥0.1 mV in at least two contiguous electrocardiogram leads (≥0.2 mv in V2,V3) and (3) an increase in the serum level of cardiac biomarkers. The definition of UA required typical chest discomfort lasting more than 20 minutes within the 24 hours preceding hospitalization and representing a change in the usual pattern of angina or pain: occurring with a crescendo pattern, being severe and described as a frank pain. The diagnosis of UA might be new or based on dynamic ST segment or T-wave changes in at least two contiguous leads.^11^ SCD was natural death with cardiac etiology preceded with sudden loss of consciousness within 1 hour of the onset of an acute change in cardiovascular status. Stroke was confirmed if the participant had new neurological deficits that persisted for >24 hours.

### Statistical analysis

Data entry was done using Epi Info, version 6 (Centers for Disease Control, Atlanta, GA). Data were analyzed by SPSS software, version 15 (SPSS Inc, Chicago, IL). Normality of data was checked with Kolmogorov–Smirnov test. The sum of individual follow-up times were used to calculate person-years until the last telephone call interview, incident IHD, non-cardiovascular death, emigration or any other loss to follow-up. Continuous variables were presented with mean ± SD and were compared with Student’s *t* test and discrete variables were compared with using chi-square or Fisher exact test (if necessary). *P* value less than 0.05 was considered statistically significant.

Cox proportional-hazard models were used to estimate hazard ratios (HRs) for future cardiovascular events. Two model adjustments were done, one for age and smoking and the other for age, smoking and menopausal status. At the beginning, 3255 women entered the study, but after 9 years of follow-up, the event data of 2784 women were statistically analyzed (471 missing data).

## Results

In ICS, 2784 women were followed for 9 years. The mean age of women at entry was 50.2±11.3 years (range: 35-91 years) and 41.6% (1154) were post menopause at the beginning of study.

After 9 years follow-up 265 cardiovascular event occurred. One hundred ninety nine of events (75.1%) were IHD and 66 events (24.9%) were stroke. Among IHD events 15 (7.5%) were fatal MI, 30 (15.07%) were nonfatal MI, 134 (67.3%) were unstable angina and 20 (10.05%) were SCD. From 66 cases of stroke, 13(19.7%) were fatal ischemic stroke and 53 (80.3%) were nonfatal stroke.

Absolute risk for incidence of ischemic heart disease and ischemic stroke for women was 889 (774-1022) and 295 (232-375) in 100 000 person-years respectively.

In Table 1, the mean quantitative values of basic cardiovascular RFs in women with and without a cardiovascular event (CV event) are presented. Women with a CV event were 8 years (in average) older than woman without CV event (57.6 ± 10.9 vs. 49.5 ± 11.1, respectively, *P* value <0.001). The mean value of cardiovascular RFs were significantly higher in women with CV event, except for HDL-C and apo-A.

**Table 1:** Comparison of basic characteristics of risk factors in women with and without cardiovascular disease events

**Women**	**Without Event** ^a^ **n = 2519**	**With Event** ^a^ **n = 265**	***P*** ** value**	**Total** ^a^ **n = 2784**
Age at entry	49.5 ± 11.1	57.6 ± 10.9	<0.001	50.2 ± 11.3
Systolic blood pressure	120.6 ± 20.7	136.7 ± 24.7	<0.001	122.1 ± 21.6
Diastolic blood pressure	77.9 ± 11.8	85.5 ± 13.5	<0.001	78.6 ± 12.2
Fasting blood sugar	87.9 ± 30.8	101.38 ± 46.95	<0.001	89.11± 32.77
BMI	27.7 ± 4.6	29.06 ± 5.16	<0.001	27.8 ± 4.7
Waist circumference	96.4 ± 12.7	100.11 ± 13.32	<0.001	96.7 ± 12.8
Waist to hip ratio	0.93 ± 0.08	0.96 ± 0.08	<0.001	0.93 ± 0.08
Triglyceride^b^	163.6 ± 96.9	200.3 ± 116.5	<0.001	187.9 ± 99.4
Total cholesterol	218 ± 51.4	237.4 ± 57.3	<0.001	219.7 ± 52.2
HDL-C	48.4 ± 10.5	48.2 ± 10	0.731	48.4 ± 10.4
LDL-C	132.7 ± 42.8	144.3 ± 46.7	<0.001	133.7 ± 43.2
TC/HDL-C	4.7 ± 1.3	5.1 ± 1.5	<0.001	4.7 ± 1.4
apo A^c^	158.6 ± 39.6	158.8 ± 46.1	0.974	158.6 ± 40.3
apo B^c^	120 ± 32.6	137.3 ± 37	<0.001	123 ± 33.9
apo B/apo A^c^	0.78 ± 0.21	0.90 ± 0.28	<0.001	0.8 ± 0.23

Abbreviations: CVD, cardiovascular disease; TC, total cholesterol; HDL-C, high-density lipoprotein-cholesterol; LDL-C, low-density lipoprotein-cholesterol; BMI, body mass index. ^a^ mean ± standard deviation. ^b^ use log transform. ^c^n = 406 (without event), 82 (with event).

According to Table 2, prevalence of all CV RFs except for overweight, low HDL-C and low apo-A were significantly more in women with event.

**Table 2:** Distribution of risk factors in women with and without cardiovascular disease

**Women**	Without Event* **n = 2519**	With Event* **n = 265**	***P*** ** value**	Total* **n = 2784**
Diabetes^a^	9.8% (248)	24.2% (64)	<0.001	11.2%(312)
Current Smoker^b^	3.0% (76)	6.41% (17)	0.006	3.3% (93)
HTN^c^	27.9% (702)	59.2% (157)	<0.001	30.9% (859)
Waist circumference >80	89.9% (2264)	94.3% (250)	0.016	90.3% (2515)
Overweight^d^	40.7% (1025)	41.1% (109)	0.896	40.7% (1134)
Obesity^e^	30.0% (756)	37.7% (100)	0.012	30.7% (856)
Dyslipidemia^f^	91.1% (2257)	96.6% (253)	0.001	91.6% (2510)
High triglyceride	57.6% (1451)	77.0% (204)	<0.001	59.4% (1655)
High TC	62.7% (1580)	78.5% (208)	<0.001	64.2%(1788)
Low HDL-C	58.9%(1483)	62.3% (165)	0.289	59.2% (1648)
High LDL-C	53.4% (1345)	70.2% (186)	<0.001	55.0% (1531)
Low apo A**	0.5% (2)	2.4% (2)	0.133	0.8% (4)
High apo B**	15.3%(62)	40.2% (33)	<0.001	19.5% (95)
High apo A/apo B**	31.1% (125)	54. 3% (44)	<0.001	35.0% (169)
Menopausal	38.7% (972)	68.9% (182)	<0.001	41.6% (1154)

Abbreviations: CVD, Cardiovascular disease; TC, total cholesterol; HDL-C, high-density lipoprotein-cholesterol; LDL-C, low-density lipoprotein-cholesterol; HTN, Hypertension. ^a^Diabetes mellitus: fasting blood glucose ≥126 mg/dL or receiving anti-diabetic agents. ^b^Current Smoker: Participants who used at least one cigarette per day. ^c^Hypertension: systolic blood pressure ≥140 mm Hg, diastolic blood pressure ≥90mmHg or current treatment for hypertension. ^d^Overweight: 25 kg/m^2^≤body mass index <30 kg/m^2^. ^e^Obesity: body mass index ≥30 kg/m^2^. ^f^Dyslipidemia: LDL-C ≥130 mg/dL, total cholesterol ≥200 mg/dL, triglyceride ≥150 mg/dL, HDL-C <40 mg/dL in men and <50 mg/dL in women. * proportion (%). **n=406 (without event), 82 (with event).

Table 3 indicates the crude and adjusted HRs of RFs for CV event. The highest adjusted & crude HR was for HTN (3.76 crude HR, 2.61 adjusted HR, *P *< 0.001). After HTN, other RFs with decreasing order of crude HR were: diabetes mellitus (2.92, *P *< 0.001), high TG (2.46, *P *< 0.001), high LDL-C (2.05, *P *< 0.001), abdominal obesity (1.87, *P *< 0.001) and obesity (1.34, *P *< 0.05). After adjustment for age, smoking and menopause status, the adjusted HR for abdominal obesity did not remain significant. Additionally, Crude and adjusted HR were not significant for low HDL-C (1.15 and 1.26 respectively) (Table 3).

**Table 3:** Crude and adjusted Hazard ratio (95% CI) of risk factors for cardiovascular events

Women	Crude	Adjusted*	Adjusted**
HTN	3.76† (2.90,4.88)	2.61† (1.97,3.45)	2.56† (1.93,3.95)
Diabetes	2.92† (2.14,3.98)	2.46* (1.79,3.39)	2.43* (1.76,3.35)
High TG	2.46† (1.83,3.31)	2.07† (1.53,2.81)	2.02† (1.49,2.74)
High LDL-C	2.05† (1.56,2.07)	1.59† (1.02,2.11)	1.59† (1.20,2.11)
Abdominal obesity	1.87† (1.10,3.21)	1.74 (1.00,3.01)	1.71 (0.99,2.96)
Obesity	1.34* (1.05-1.72)	1.48* (1.15-1.91)	1.49* (1.16-1.92)
Low HDL-C	1.15 (0.89,1.49)	1.25 (0.95,1.63)	1.26 (0.96,1.65)

Abbreviations: TG, triglyceride; HDL-C: high density lipoprotein-cholesterol; LDL-C: low density lipoprotein-cholesterol; HTN, Hypertension. * Adjusted model including age and smoking. ** Adjusted model including age, smoking and menopause. P-value: * *P *< 0.05; § *P *< 0.01; † *P *< 0.001.

## Discussion

In ICS, an ongoing population based cohort in Eastern Mediterranean region that women represented more than half of total study group, we found that HTN, DM, high TG, high LDL-C and obesity were the most important predictors for CVD events in women while abdominal obesity had modest relationship and low HDL-C did not show any association.

In ICS, the mean age of women with CV event was about 58 ± 11 years, approximately 8 years more than mean age of women without event. These data are consistent with those observed in INTERHEART study which indicated the mean age of Middle East women with MI in the study was 57 years (range 50-65), that was 11 years younger than western European women with MI (mean age: 68 years).^12^ This young age of CVD incidence, show the importance of evaluating different cardiovascular RFs in Middle East women in earlier age.

It is estimated that the prevalence of CVD in diabetic women is twice as common as non-diabetics and rate of hospitalization is four times in them.^13^ In ICS about 24% of women with CV event were diabetic compared to 10% in women without event (*P* < 0.001) and the risk of CVD events in diabetic women was about 2.5 times more than non-diabetics. These data were similar to those of Framingham^12,14^ which indicated the prevalence of CVD in diabetic women was three times compared with non-diabetics.^14^

HTN was the most powerful RF for CVD events in women in our study. In ICS All women with fatal MI had HTN and in overall about 60% of women with CV event were hypertensive. The risk of CVD in hypertensive women increases about 2.5 times compared with non-hypertensive’s. The study carried out by Stangl et al similarly suggested the risk of incident CVD events to be approximately 3.5 times higher in hypertensive women.^15^

Smoking alter estrogen metabolism and causes premature menopause, change of lipid profile and increased risk of IHD. According to US Department of Health and Human Services,^16^ age of death from CVD was about 14.5 years younger in female smokers compared to age-matched nonsmokers. Additionally, several studies indicated that smoking increase the chance of CVD.^17-20^ Although smoking was not so prevalent in ICS women (only 3.3%) but in line with mentioned studies was significantly more prevalent in women with a CV event.

In different studies varied types of dyslipidemia have been introduced as strong predictors of CVD.^21-25^ In Women’s Health Study,^23^ among different types of hyperlipidemia, high non-HDL-C and apoB-100 was reported to have the highest HR for CV events in women (2.51, 2.50 respectively). Others reported that hypertriglyceridemia is better predictor of CHD in women.^22,24,26^ In another study the relative risk (RR) of different types of dyslipidemia on cardiac events in women was reported and the highest RR was for apoB (RR: 4.1), after that were high LDL-C, low HDL-C and high TG with RRs of 3.1, 2.6 and 1.9, respectively.^25^ In ICS, more than 95% of women with CVD event had dyslipidemia and among different types of dyslipidemia the highest crude and adjusted HR for CV event was for high TG and after that was high LDL-C. Additionally, Low HDL-C was not important RFs for CV event in women in our study. Unexpectedly, low HDL-C was significantly more common in premenopausal women (61.9% vs. 53.7%, *P *< 0.001). The old age of women in our study and more prevalence of low HDL-C in premenopausal women can explain why low HDL-C was not significant RF for CVD.

In ICS the mean value of BMI, waist circumference and waist to hip ratio were significantly higher in women with CV event (for all *P *< 0.001). Abdominal obesity in women was significant RF but after adjustment for other RFs did not remain significant and this may be explained by the difference between Iranian cut-off WC value and international recommended value^27^ while obesity was a RF for CV events after adjustment for other confounders.

The main limitation of the present study was a remarkable loss-to-follow-up, similar to other cohort studies, however the characteristics of subjects lost to follow-up were not so different that could significantly affect the internal validity of the study. Additionally, behavioral RFs (except smoking) such as level of exercise and type of diet did not mention in this study and further research should be conducted aimed to evaluating these RFs among Iranian women.

In summary, we evaluated CVD RFs among Iranian women which showed that HTN, diabetes mellitus, high TG are strong predictors for CV events in Iranian women. As these RFs can be prevented, health professionals must be aware of them to make preventive public health strategies and also enhance knowledge of women about CVD RF threats. Moreover, this study according to best of our knowledge would help to provide validated data derived from Eastern Mediterranean region regarding to women health.

## Acknowledgements

This cohort study was conducted by ICRC affiliated with the Isfahan University of Medical Sciences, Isfahan, Iran. We would like to express our appreciation to the Isfahan Provincial Health Center, Najaf-Abad Health Office and Arak University of Medical Sciences and also sincere appreciation to the ICS team, especially to Mrs. Mansoureh Boshtam, Mr. Hossein Balouchi, Dr. Hossein Heidari and Dr. Ahmad Bahonar for their technical assistance.

## Competing interest

None.

## Ethical approval

The Ethics Committee of ICRC, a World Health Organization-collaborating center, approved study protocol and all subjects gave written informed consent.
